# Supplementary material for: Sexual and reproductive health challenges among street adolescents in Sylhet city, Bangladesh: A cross-sectional study
Source: PLoS One. 2026 Jan 14;21(1):e0340865. doi: 10.1371/journal.pone.0340865 (PMC12803461; doi:10.1371/journal.pone.0340865)
Supplement: S1 File — (DOCX) [file pone.0340865.s001.docx]

**Questionnaire (English)**

**Study Title: Sexual and Reproductive Health Related Behaviors among Street Adolescent**

Sample ID:

**Name of the respondents: ........................................................................................**

**Phone Number:**

**Email Address:**

Date of data collection: ______/______/____________

**Starting time of interview** (24 hours clock) Hours: Minutes :

**Section 1: Socio-demographic characteristics**

| SL No. | Questions | Response | Code |
| --- | --- | --- | --- |
| 01 | What is your age in years? | _______years |  |
| 02 | What is your gender? | 1.Male  2.Female  3.Transgender |  |
| 03 | What is the religion you follow? | 1. Islam  2. Hindu  3. Christian  4. Buddhist |  |
| 04 | What is your marital status? | 1=Married  2=Unmarried  3=Divorced  4=Separated  5=widow |  |
| 05 | Are you currently enrolled in any educational institute? | 1= Yes  2= No |  |
| 06 | If yes, in which group of school? |  |  |
| 07 | What is the educational status of your father? | 1= No formal education  2= Primary  3= Secondary  4= Higher secondary  5= Others |  |
| 08 | What is the educational status of your mother? | 1= No formal education  2= Primary  3= Secondary  4= Higher secondary  5= Others |  |

| 09 | What is your father’s occupation? | 1= Unemployed  2= Day labor  3= Business  4= Agricultural worker  5= Service holder  99= Others |  |
| --- | --- | --- | --- |
| 10 | What is your mother’s occupation? | 1= Unemployed  2= Home maker  3= Day labor  4= Service holder  5= Agricultural worker  99= Others |  |
| 11 | What is your current relationship status with yours family member? | 1=Living with parents  2= Living with mother  3= Living with father  4= Living with sibling  5=Alone |  |
| 12 | What are the types of your family? | 1=Nuclear  2=Single parents families  3=Extended families  4= Childless families  5=Step families  6= Grandparents families |  |
| 13 | How many siblings you have? | 1= No siblings  2= One  3= Two  4= Three  99= Others |  |
| 14 | What is your approximate income per day?? | in TK |  |
| 15 | What is your earning source ? |  |  |
| 16 | What is your approximate expense per day? | In TK |  |

**Part 2: Question related to sexual and reproductive health**

| 17 | Do you ever been suffered from any sexual disease? | 1= Yes  2= No |  |
| --- | --- | --- | --- |
| 18 | If yes, which one? |  |  |
| 19 | Have you ever had sexual intercourse? [not only with girl/boyfriend] | 1= Yes  2= No |  |
| 20 | Can pregnancy be prevented by using contraception? | 1= Yes  2= No |  |
| 21 | If yes, which one you use/ take? |  |  |
| 22 | Have you ever been conceive? | 1= Yes  2= No |  |
| 23 | Have you ever had an abortion? | 1= Yes  2= No |  |
| 24 | Did you face any problems in your sexual life due to SRH problems? | 1= Yes  2= No |  |
| 25 | If yes, what is the problem? |  |  |

**Part 3:Question related to pubertal changes**

| SL No. | Questions | Response | Code |
| --- | --- | --- | --- |
| 26 | In case of male ,When your pubertal period is start? | In years |  |
| 27 | Have you experienced wet dreams? | 1.Yes  2.No |  |

| 28 | In case of female , Did your menses start? | 1.Yes  2.No |  |
| --- | --- | --- | --- |
| 29 | If yes, what was your age at menarche? | In years |  |
| 30 | what was your experience? | 1. unpleasant 2. pleasant 3. no feeling |  |
| 31 | Did you receive any information regarding menstruation before ? | 1.Yes  2.No |  |
| 32 | If yes, then what was your source of information? | 1.Parents  2.Peer  3.Mass media  99. Others |  |
| 33 | What type of absorbent do you use during menstruation? | 1. Sanitary Pad   2 .Cloths  99. Others |  |
| 34. | What are the reasons for not using sanitary pads? | 1.No reason  2.Difficulty in discard  3.Costly  4.Don’t know about it  5.Don’t feel comfortable in it |  |
| 35 | Do you have any bathroom in your residing? | 1.Yes  2.No |  |
| 36 | Have you ever experienced any form of abnormal sexual behavior? | 1.Yes  2.No |  |
| 37 | If yes, then what was the nature of abuse? | 1.Touching  2.Sexual intercourse  3.Forced to show naked body  99. Others |  |
| 38 | How many times did the abuse occur? |  |  |

**Part 4: Question related to sexual and reproductive health seeking behaviors**

| 39 | Is your menses? | 1. Regular 2. Irregular |  |
| --- | --- | --- | --- |
| 40 | Do you have any others reproductive health problems? |  |  |
| 41 | Have you ever been any treatment for your reproductive health problems from any doctors/healthcare services? | 1.Yes  2. No |  |
| 42 | If yes, from whom you took the treatment? | 1.Doctor  99.Others |  |
| 43 | Where are the SRH services available? | 1. Govt. hospital  2. Private hospital  3.Community hospital  4 .NGOs  5.Medicine shops/ pharmacy  99. Others |  |
| 44 | Do you know about transmission of STDs? | 1. Yes 2. No |  |
| 45 | If yes, how it transmit? |  |  |
| 46 | Do you know about the risk factors of STDs? | 1.Yes  2. No |  |
| 47 | If yes, what are the risk factors of STDs? | 1. Having unprotected sex 2. Having sexual contact with multiple partner 3. Having a history of STIs 4. Being forced to engage in sexual activity 5. Use of injecting drugs   99 .others |  |
| 48 | Have you ever taken SRH services? | 1.Yes  2. No |  |
| 49 | If no ,what are the difficulties to get easy access to SRH services? | 1. Socio cultural barrier 2. Lack of knowledge 3. Lack of sources 4. Economical barrier 5. Lack of adolescent friendly services 6. Lack of confidentiality 7. Due to long distance health care center 8. Embarrassment 9. Long waiting time   99. Others |  |
| 50 | 74.If ‘Yes’, how was the attitude of healthcare providers when taking services? | 1.Helpful  2. Not helpful  3. As usual |  |

**Part 5: Question related to abnormal sexual behavior:**

| SL No. | Questions | Response | Code |
| --- | --- | --- | --- |
| 51 | Have you ever been sexually abused? | 1.Yes  2. No |  |
| 52 | If yes, how were you abused? | 1. Physical  2. Psychological  3. Sexual |  |
| 53 | By whom have you been abused? | 1. Parents  2. Relatives  3. Peers  4. Others (Please specify….) |  |

**Thank you for your contribution in providing feedback in this questionnaire form**
